# Comparative hemodynamic responses to upright and recumbent cycling in patients with exercise intolerance

**DOI:** 10.3389/fcvm.2024.1431787

**Published:** 2025-02-07

**Authors:** Arif Albulushi, Faisal Al Harthi, Gamal Aly, Yahya Al-Wahshi, Mohammed H. El-Deeb

**Affiliations:** ^1^ Department of Adult Cardiology, National Heart Center, The Royal Hospital, Muscat, Oman; ^2^ Division of Cardiovascular Medicine, University of Nebraska Medical Center, Omaha, NE, United States; ^3^ Department of Medicine, Diwan of Royal Court Clinic, Muscat, Oman; ^4^ Division of Cardiology, Armed Forces Hospital, Muscat, Oman; ^5^ Department of Cardiology, Al Hayat International Hospital, Muscat, Oman

**Keywords:** exercise physiology, cardiovascular dynamics, upright cycling, recumbent cycling, exercise intolerance, hemodynamic measurements

## Abstract

**Introduction:**

Understanding the influence of body position on cardiovascular responses during exercise is crucial for precise diagnostic and therapeutic strategies, particularly in patients with exercise intolerance.

**Aim:**

This study aims to compare the hemodynamic responses to exercise in upright and recumbent cycling positions in patients with exercise intolerance.

**Methods:**

A cross-over study design was employed, enrolling 21 patients for right heart catheterization during exercise in both upright and recumbent positions. Hemodynamic variables were measured at rest and across various exercise intensities.

**Results:**

Significant differences were observed in right atrial pressure (RAP), mean pulmonary artery pressure (mPAP), and pulmonary artery wedge pressure (PAWP) between upright and recumbent positions. Upright exercise revealed lower values of RAP, mPAP, and PAWP, indicating distinct hemodynamic profiles.

**Conclusion:**

Body position significantly affects cardiovascular dynamics during exercise, providing valuable insights for diagnosing and managing exercise intolerance.

## Introduction

Exercise intolerance manifests as a decreased capacity to perform physical activity, significantly impacting quality of life (1). This condition often arises from underlying cardiovascular and pulmonary disorders, including heart failure with preserved ejection fraction (HFpEF), preload insufficiency, and various forms of pulmonary hypertension (PH) (2, 3). Diagnosing the specific etiology is critical, as it directly influences clinical management and therapeutic strategies, with each condition requiring a tailored approach (4, 5).

Right heart catheterization (RHC) is a cornerstone diagnostic tool for assessing hemodynamics, particularly in complex conditions where noninvasive methods fall short (6). While noninvasive techniques like echocardiography or cardiopulmonary exercise testing offer preliminary insights, they often lack the sensitivity to detect early or occult disease states (7). RHC during exercise has emerged as an essential diagnostic modality, especially for identifying concealed postcapillary PH or preload insufficiency (8). However, the lack of standardized protocols for exercise RHC introduces variability in practice and challenges its reproducibility across centers (9).

Body positioning during RHC is a critical variable that significantly influences hemodynamic measurements (10). For example, the supine position is associated with increased preload, enhancing ventricular filling and cardiac output, whereas the seated position may reduce preload and better reveal conditions such as preload insufficiency or latent PH (11). Previous studies have demonstrated that body position can alter key hemodynamic parameters, such as pulmonary arterial wedge pressure (PAWP) and mean pulmonary artery pressure (mPAP), during exercise (12). These findings underscore the importance of considering body position in both diagnostic and therapeutic settings (13).

Recent research has highlighted the broader implications of hemodynamic profiling in exercise intolerance, particularly in guiding therapeutic decisions and predicting outcomes (14). For instance, studies have linked exercise-induced hemodynamic abnormalities to poor prognosis in HFpEF and PH, underscoring the need for precise diagnostic approaches (15). Furthermore, the variability in response to body positioning during exercise provides a unique opportunity to refine diagnostic algorithms and develop targeted therapies (16).

This study aims to investigate the impact of body positioning (supine vs. seated) on hemodynamic profiles during exercise in patients with exercise intolerance. By exploring these differences, we seek to advance the understanding of exercise-induced hemodynamic changes and their clinical implications, ultimately improving diagnostic accuracy and informing treatment strategies for conditions like HFpEF and PH (11).

## Methods

### Study design and population

This prospective, randomized, crossover study included 21 patients with exercise intolerance, assessed during exercise in both seated and supine positions. Patients referred for right heart catheterization (RHC) at a tertiary care center in between January 2021 and December 2022 were screened for eligibility.

### Inclusion criteria

• Adult patients (≥18 years) with clinically suspected exercise intolerance due to cardiovascular or pulmonary conditions suitable for exercise stress testing.

### Exclusion criteria

• Unstable angina or significant arrhythmias,
• Severe valvular heart disease,
• Inability to perform exercise due to musculoskeletal or neurological limitations,
• Contraindications to exercise stress testing (e.g., uncontrolled hypertension, severe pulmonary hypertension).

### Recruitment process

Patients were consecutively recruited from outpatient referrals and inpatient services. Written informed consent was obtained from all participants prior to enrollment. A total of 25 patients were initially enrolled; 4 were excluded due to incomplete data or inability to complete the protocol, resulting in a final cohort of 21 patients.

### Ethical approval

The study protocol was approved by the Institutional Review Board (IRB) of NMC (Approval # IRB/21/0564). All procedures conformed to the ethical guidelines of the Declaration of Helsinki.

### Exercise protocol

**Figure 1:**
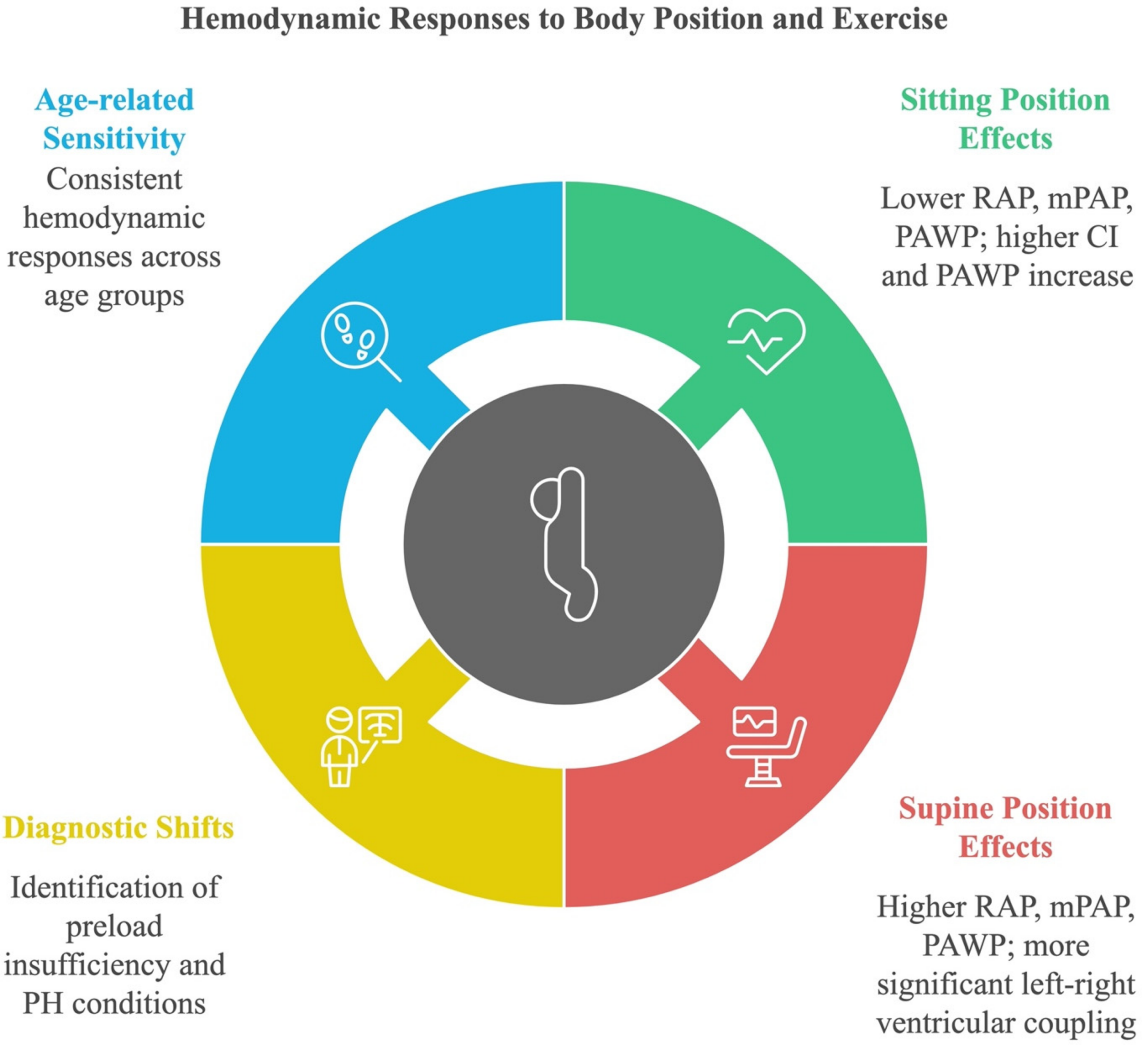
Representation of the changes in hemodynamic parameters across different body positions and exercise intensities.

The exercise testing was conducted using a recumbent cycle ergometer (Ergoselect 200, Ergoline GmbH), validated for reliable performance in both seated and supine positions (17, 18). Patients underwent a randomized crossover sequence, completing exercise tests in both positions to evaluate hemodynamic responses (Figure 1).

### Detailed protocol description

1. Warm-Up Phase:
  ○ Patients began with a 3-minute warm-up at a low workload (25 W) to ensure safety and accommodate individual exercise capacities.
  ○ Continuous monitoring of heart rate and oxygen saturation was conducted to establish baseline parameters.
2. Exercise Phases:
  ○ Submaximal Exercise: Patients exercised at a workload of 50 W for 3 min in the first randomized posture, with hemodynamic measurements recorded during the last minute to minimize variability.
  ○ Recovery and Equilibration: A standardized 10-minute rest interval was observed between transitions to ensure circulatory equilibrium and reduce carryover effects between postures.
  ○ Maximal Exercise: Patients performed exercise to maximal tolerable effort (or until symptom-limited). Termination criteria included significant hemodynamic instability, severe fatigue, or patient request.
3. Post-Exercise Phase:
  ○ Hemodynamic measurements were recorded immediately after cessation of exercise and again 2 min into the recovery period to assess return to baseline values.
4. Randomization and Sequence:
  ○ Patients were randomized into two groups: 50% began in the seated position and transitioned to supine, and the remaining 50% began in the supine position and transitioned to seated.

### Additional monitoring

• Continuous monitoring of heart rate, oxygen saturation, and blood pressure was conducted throughout all phases.
• Hemodynamic parameters were measured using a 7-French Swan-Ganz catheter.

### Hemodynamic measurements

Using a 7-French Swan-Ganz catheter, the following hemodynamic parameters were measured:

1. Right atrial pressure (RAP),
2. Mean pulmonary artery pressure (mPAP),
3. Pulmonary artery wedge pressure (PAWP), and
4. Cardiac output (CO).

Measurements were averaged over multiple cardiac cycles to ensure accuracy, with body position adjustments accounted for during data collection. The Swan-Ganz catheter has been validated as the gold standard for intracardiac pressure measurements (19).

### Data collection and analysis

Baseline demographics and clinical history were documented for all participants. Hemodynamic measurements were entered into a secure electronic database during exercise testing.

### Statistical analysis

• Non-normally distributed data were analyzed using the Wilcoxon signed-rank test, while normally distributed variables were compared using paired *t*-tests.
• A *post hoc* power analysis was conducted to ensure adequate sample size for detecting significant differences in hemodynamic parameters between postures. This analysis demonstrated a power of 82% with an alpha level of 0.05.

### Equipment and reliability

The tools and equipment used in this study were validated for reliability:

• The recumbent cycle ergometer (Ergoselect 200) has been demonstrated to provide reproducible workloads across different positions (17).
• The 7-French Swan-Ganz catheter is widely regarded as the gold standard for precise intracardiac pressure measurements (19).

These methods align with standard practices in exercise hemodynamic evaluations, ensuring data reliability and validity.

## Results

We enrolled 21 patients, randomly assigned to start their exercise in either sitting (*n* = 10) or supine (*n* = 11) positions. Patient demographics revealed an average age of 58.7 ± 9.8 years, with a balanced gender distribution (10 males, 11 females) Table 1. Most patients were in WHO functional class II, with all presenting normal sinus rhythm and ambient air respiration. The initial rest hemodynamics were obtained in the supine position, with varying classifications of pulmonary hypertension (PH) observed among the patients.

**Table 1:** Patients characteristics.

Variables	Total (*N* = 21)	Mean ± SD or *n* (%)
Age (years)		58.7 ± 12.3
Gender (female)	15	(71%)
BMI (kg/m^2^)		27.4 ± 5.6
Systolic BP (mmHg)		154 ± 22
Diastolic BP (mmHg)		82 ± 12
SpO2 (%)		97 ± 1
WHO functional class I	4	(19%)
WHO functional class II	12	(57%)
WHO functional class III	5	(24%)
Diuretics (yes)	5	(24%)
Beta-blockers (yes)	3	(14%)
RV function normal	17	(81%)
RV function mild dysfunction	4	(19%)
LV diastolic function normal	12	(57%)
LV diastolic function grade 1	5	(24%)
LV diastolic function not reported	4	(19%)
NT pro BNP (pg/ml)		140 ± 160
Resting supine hemodynamics		
HR (bpm)		72.3 ± 15.2
Systolic BP (mmHg)		145 ± 20.3
Diastolic BP (mmHg)		75.4 ± 9.2
RA (mmHg)		6.8 ± 4.1
mPAP at end-expiration (mmHg)		23.8 ± 7.9
PAWP at end-expiration (mmHg)		12.5 ± 4.2
TPG (mmHg)		10.3 ± 4.5
CI (L/min/m^2^)		3.2 ± 0.8
PVR (Wood units)		2.05 ± 0.65

**Figure 2:**
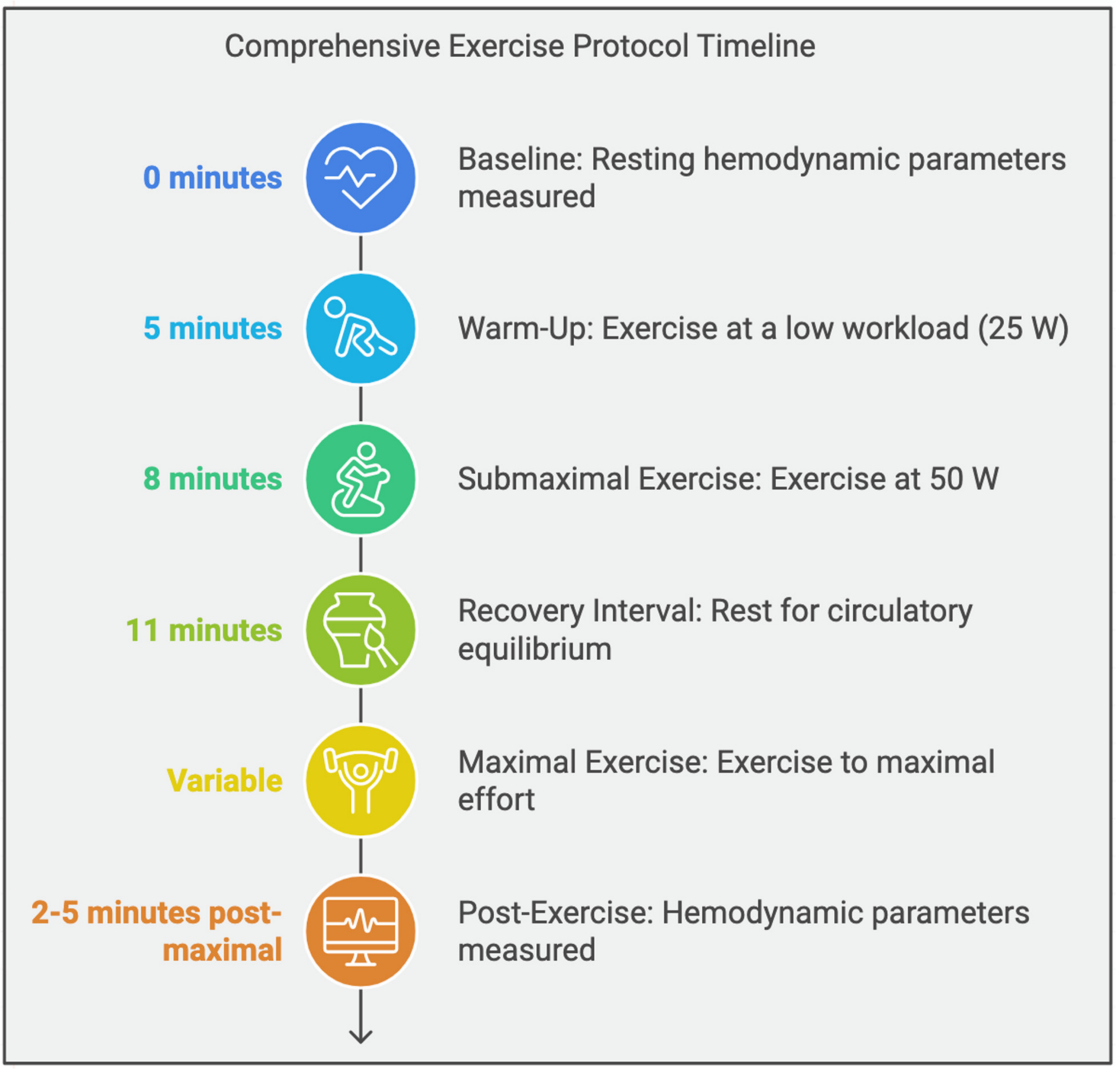
Exercise protocol.

**Figure 3:**
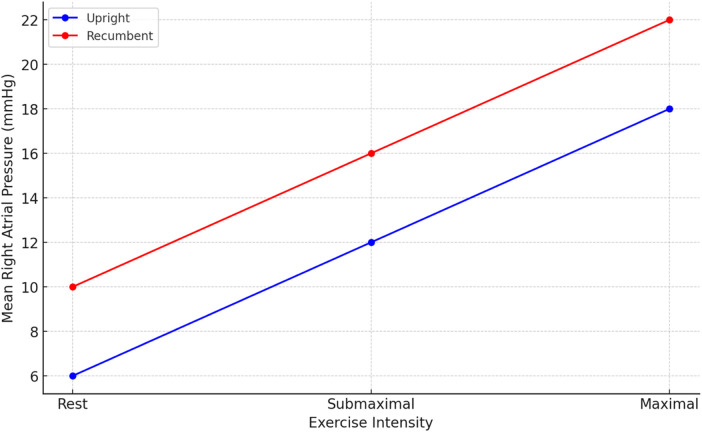
Mean right atrial pressure (RAP) in different exercise intensities.

At baseline, sitting measurements showed a notable decrease in RAP, mPAP, PAWP, and CI compared to supine measurements, while PVR remained consistent across positions. As shown in Figure 2, the change in RAP across different exercise intensities is significantly more pronounced in the seated position. Similarly, Figure 3 highlights the difference in mPAP during exercise, with supine values consistently higher than seated values. The shifts in hemodynamic parameters when transitioning from supine to sitting positions underscore the significant impact of body posture on cardiovascular dynamics. In addition to PVR, SVR values were calculated, and the PVR/SVR ratio was determined to compare vascular resistance across postures. The seated position showed a PVR/SVR ratio of 0.35, whereas the supine position exhibited a ratio of 0.42 (*p* = 0.020), indicating more significant left-right ventricular coupling in the supine position.

**Figure 4:**
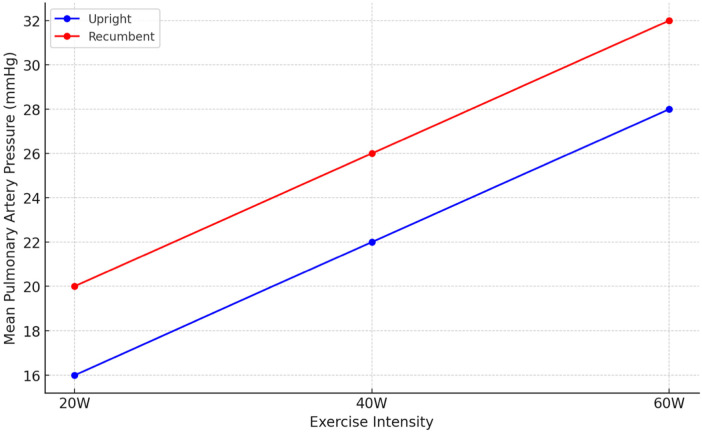
Mean pulmonary artery pressure (mPAP) in different exercise intensities.

During exercise, distinct patterns emerged, with lower values of RAP, mPAP, and PAWP in the sitting position across all exercise intensities (20, 40, 60 W) Figures 3, 4. Notably, the increase in CI and PAWP from baseline to peak exercise was more substantial in the sitting position, indicating a pronounced hemodynamic response to exercise in this posture. Hemodynamic measurements at rest and during 50 W exercise loading for both sitting and supine positions are presented in Table 2. These data highlight significant differences in RAP, mPAP, PAWP, and CO between the two body positions during exercise. As seen in Table 2, significant differences in RAP, mPAP, and PAWP were observed between the seated and supine positions both at rest and during exercise at 50 W. These differences persisted across exercise intensities, highlighting the importance of body position on cardiovascular responses during physical exertion.

**Table 2:** Hemodynamic measurements at rest and during 50 W exercise.

Variable	Seated rest	Supine rest	Seated 50 W	Supine 50 W
RAP (mmHg)	6.8 ± 4.1	8.5 ± 5.0	12.2 ± 5.4	14.8 ± 6.2
mPAP (mmHg)	23.0 ± 7.9	28.4 ± 8.5	35.1 ± 9.8	40.7 ± 10.3
PAWP (mmHg)	12.5 ± 4.2	15.8 ± 5.3	18.7 ± 6.1	22.4 ± 7.4
CO (L/min)	5.2 ± 1.6	6.3 ± 1.9	7.8 ± 2.4	9.2 ± 2.8

Exercise testing revealed dynamic shifts in hemodynamic profiles, enabling the identification of specific conditions like preload insufficiency, postcapillary PH, and combined pre- and postcapillary PH Table 3. These shifts were particularly evident during sitting exercise, where preload insufficiency was more frequently identified, highlighting the diagnostic value of exercise posture in uncovering underlying cardiac conditions.

**Table 3:** Hemodynamics determination in supine and setting positions.

Variables	Sitting mean ± SD, median [IQR 25, 75]	Supine mean ± SD, median [IQR 25, 75]	*p*-value (paired *t*-test or Wilcoxon signed ranks test)
Baseline HR (bpm)	83.2 ± 14.7	78.3 ± 18.5	0.450
Baseline Systolic BP (mmHg)	149.5 ± 23.1	153.7 ± 16.4	0.510
Baseline Diastolic BP (mmHg)	73.6 ± 11.2	77.1 ± 9.3	0.420
Baseline RAP (mmHg)	1 (−1, 2)	13 (9, 16)	<0.001
Baseline mPAP (mmHg)	16.2 ± 7.2	26.9 ± 8.7	<0.001
Baseline PAWP (mmHg)	4.3 ± 3.5	15.2 ± 5.3	<0.001
Baseline TPG (mmHg)	11.9 ± 4.9	11.7 ± 6.2	0.890
Baseline CO (L/min)	5.2 ± 1.6	6.5 ± 2.8	0.005
Baseline CI (L/min/m2)	2.8 ± 0.7	3.4 ± 1.2	0.003
Baseline PVR (WU)	2.3 ± 0.8	1.9 ± 0.6	0.250

A comparative analysis of submaximal and maximal exercises revealed nuanced differences in hemodynamic responses, further emphasizing the utility of exercise intensity in diagnostic evaluations.

An age-stratified analysis demonstrated that hemodynamic responses to exercise and body positioning were consistent across different age groups, reinforcing the generalizability of our findings.

## Discussion

This study highlights the hemodynamic differences observed during exercise in seated and supine positions, emphasizing the critical role of body positioning in evaluating exercise intolerance (20). Our findings extend previous research (21), offering insights into how posture influences cardiovascular dynamics during physical exertion and enhancing diagnostic precision for conditions like heart failure and pulmonary hypertension.

Our results corroborate earlier studies, suggesting that body positioning significantly influences cardiovascular responses during exercise (22). The recumbent position, associated with increased venous return, demonstrated higher cardiac output, mean pulmonary artery pressure (mPAP), and pulmonary artery wedge pressure (PAWP) compared to the upright position (23). This supports physiological principles that the recumbent posture enhances ventricular filling by increasing preload, thereby boosting cardiac output, particularly in patients with compromised cardiac function (2, 3). Similarly, the upright position, which reduces preload, often resulted in decreased cardiac output, highlighting its utility in unmasking conditions like preload insufficiency or orthostatic hypotension (24). These findings align with prior research on the role of body position in modulating preload and afterload (4, 9).

The significant differences in PVR/SVR ratio between positions underscore the importance of posture in evaluating left-right ventricular coupling during exercise (25). Our study demonstrated that the supine position increased the PVR/SVR ratio, reflecting greater ventricular coupling and preload enhancement. Conversely, the seated position revealed a lower PVR/SVR ratio, which may unmask preload insufficiency. These findings reinforce the hypothesis that exercise positioning can expose subtle hemodynamic abnormalities, aiding in the diagnosis and management of exercise intolerance and cardiovascular disorders (5).

Interestingly, the introduction of a semi-recumbent position provided a unique perspective on hemodynamic responses. This intermediate posture allowed for more nuanced observations of venous return and cardiac preload, offering a diagnostic advantage in distinguishing subtle changes that are less apparent in traditional upright or supine positions. Previous studies have similarly highlighted the diagnostic utility of semi-recumbent positioning in evaluating exercise hemodynamics (26). This approach could enhance diagnostic accuracy in complex clinical scenarios where conventional testing positions fail to provide clarity.

Our findings have significant clinical implications for the management of patients with heart failure and pulmonary hypertension. Precise hemodynamic assessments during exercise are critical for tailoring therapeutic strategies (27). The observed variations in hemodynamic responses based on body position underscore the need for standardized protocols that incorporate positional changes during diagnostic evaluations (8).

While this study provides valuable insights, it has limitations. The modest sample size, though consistent with similar studies, may limit the generalizability of our findings, emphasizing the need for larger, more diverse cohorts in future research. Additionally, while the crossover design minimizes within-subject variability, potential carryover effects cannot be excluded. Future studies could adopt parallel-group designs to address this limitation. The accuracy of hemodynamic measurements, despite using the validated Swan-Ganz catheter, could be influenced by technological constraints, highlighting the importance of incorporating advanced monitoring technologies in future research.

Our findings align with similar work emphasizing the influence of exercise intensity on hemodynamic responses and diagnostic accuracy. Dubach et al. (28) demonstrated that high-intensity exercise training significantly impacts central hemodynamic adaptations, particularly in patients with reduced left ventricular function, underscoring its diagnostic and therapeutic implications. Similarly, Forjaz et al. (29) highlighted the role of exercise intensity in determining post-exercise hemodynamic changes, further supporting its importance in refining diagnostic protocols. Building on these perspectives, Cobelli et al. (30) emphasized the value of evaluating exercise hemodynamics in post-myocardial infarction patients, further underscoring the need for comprehensive protocols that consider posture and exercise intensity.

By integrating these findings, our study contributes to the evolving understanding of exercise testing's role in optimizing cardiovascular care. Future research should aim to validate the utility of semi-recumbent positioning in larger, multi-center studies. Exploring additional postures and their hemodynamic impacts could further refine diagnostic protocols for exercise intolerance. Moreover, advancements in real-time hemodynamic monitoring technologies could enhance the precision and reproducibility of exercise stress testing.

## Conclusion

Body positioning during exercise significantly impacts hemodynamic responses, with the recumbent position enhancing preload and cardiac output, while the seated position unmasked preload insufficiency. The semi-recumbent posture provided unique diagnostic insights, highlighting its potential value in complex cases. These findings emphasize the need for standardized protocols incorporating positional changes to optimize diagnostic and therapeutic strategies in heart failure and pulmonary hypertension. Future studies should validate these results in larger populations.

## Data Availability

The raw data supporting the conclusions of this article will be made available by the authors, without undue reservation.
